# Retrospective Study of the Epidemiology and Clinical Manifestations of *Cryptococcus gattii* Infections in Colombia from 1997–2011

**DOI:** 10.1371/journal.pntd.0003272

**Published:** 2014-11-20

**Authors:** Jairo Lizarazo, Patricia Escandón, Clara Inés Agudelo, Carolina Firacative, Wieland Meyer, Elizabeth Castañeda

**Affiliations:** 1 Internal Medicine Department, Hospital Universitario Erasmo Meoz, Cúcuta, Norte de Santander, Colombia; 2 Microbiology Group, Instituto Nacional de Salud, Bogotá, Colombia; 3 Molecular Mycology Research Laboratory, Centre for Infectious Diseases and Microbiology, Sydney Medical School – Westmead Hospital, Marie Bashir Institute for Infectious Diseases and Biosecurity, The University of Sydney, Westmead Millennium Institute, Sydney, Australia; University of California San Diego School of Medicine, United States of America

## Abstract

**Background:**

Cryptococcosis due to *Cryptococcus gattii* is endemic in various parts of the world, affecting mostly immunocompetent patients. A national surveillance study of cryptococcosis, including demographical, clinical and microbiological data, has been ongoing since 1997 in Colombia, to provide insights into the epidemiology of this mycosis.

**Methodology/Principal Findings:**

From 1,209 surveys analyzed between 1997–2011, 45 cases caused by *C. gattii* were reported (prevalence 3.7%; annual incidence 0.07 cases/million inhabitants/year). Norte de Santander had the highest incidence (0.81 cases/million/year), representing 33.3% of all cases. The male: female ratio was 3.3∶1. Mean age at diagnosis was 41±16 years. No specific risk factors were identified in 91.1% of patients. HIV infection was reported in 6.7% of patients, autoimmune disease and steroids use in 2.2%. Clinical features included headache (80.5%), nausea/vomiting (56.1%) and neurological derangements (48.8%). Chest radiographs were taken in 21 (46.7%) cases, with abnormal findings in 7 (33.3%). Cranial CT scans were obtained in 15 (33.3%) cases, with abnormalities detected in 10 (66.7%). Treatment was well documented in 30 cases, with most receiving amphotericin B. Direct sample examination was positive in 97.7% cases. Antigen detection was positive for all CSF specimens and for 75% of serum samples. *C. gattii* was recovered from CSF (93.3%) and respiratory specimens (6.6%). Serotype was determined in 42 isolates; 36 isolates were serotype B (85.7%), while 6 were C (14.3%). The breakdowns of molecular types were VGII (55.6%), VGIII (31.1%) and VGI (13.3%). Among 44 strains, 16 MLST sequence types (ST) were identified, 11 of them newly reported.

**Conclusions/Significance:**

The results of this passive surveillance study demonstrate that cryptococcosis caused by *C. gattii* has a low prevalence in Colombia, with the exception of Norte de Santander. The predominance of molecular type VGII is of concern considering its association with high virulence and the potential to evolve into outbreaks.

## Author Summary

Cryptococcosis is caused by *Cryptococcus neoformans* and *C. gattii*, with the most serious manifestation of disease being infection of the central nervous system (CNS). *C. neoformans* tends to cause disease in immunosuppressed patients, especially those infected with HIV, while *C. gattii* affects immunocompetent patients preferentially. *C. gattii* is usually endemic in tropical and subtropical areas. However, highly virulent strains have recently emerged in temperate areas, such as British Columbia in Canada and the Pacific Northwest of the United States. The Colombian national cryptococcal survey characterized the demographic, clinical manifestations and microbiological aspects of *C. gattii* cryptococcosis. An annual average incidence of infection of 0.07 cases/million inhabitants/year was determined. In contrast, in Norte de Santander the incidence reached 0.81 cases/million inhabitants/year. The national prevalence was 3.7% among all forms of cryptococcosis. Involvement of the CNS (88%) was the commonest clinical manifestation of cryptococcosis. Molecular type VGII, which is the same molecular type as described in the recent outbreaks of this mycosis, was the most prevalent. Overall, clinical *C. gattii* strains from Colombia showed great genetic diversity. This work contributes to knowledge of the global epidemiology of cryptococcosis and its clinical behavior in Colombian patients.

## Introduction

Cryptococcosis is a fungal disease that affects humans and animals and is caused by two species, *Cryptococcus neoforma*ns and *Cryptococcus gattii* [1]. *C. gattii* has been recognized as a distinct species from *C. neoformans* due to differences in the morphology of the basidia, environmental niches, multiple gene genealogies, unique patterns generated by different molecular typing techniques, inefficient crossing of species with the production of sterile progeny and a lack of genetic recombination [2]. *C. gattii* can be easily and reliably differentiated from *C. neoformans* through a simple phenotypic procedure, growth on CGB (canavanine, glycine and bromothymol blue) culture medium [3]. *C. gattii* assimilates glycine, is resistant to canavanine and changes the color of the media due to an alteration in pH when creatinine is degraded into ammonia. *C. neoformans* is not able to assimilate glycine; therefore, it does not grow on this media [3].

The two species cause different clinical manifestations and have different biological characteristics [1]. *C. neoformans* is responsible for most cases of cryptococcosis worldwide [4]. Until recently *C. gattii* was considered rare, however cryptococcosis by *C. gattii* has gained importance because of its emergence in 1999, which resulted in an outbreak on Vancouver Island and other closely related regions in British Columbia, Canada [5] and the increasing number of cases since 2004 in the Pacific Northwest of the United States [6]. Recent studies based on Multilocus Microsatellite Type (MLMT) and Multilocus Sequence Type (MLST) analyses, as well as whole genome analysis, point towards South America as a potential origin for the outbreak strains [7], [8].

Previously, it was thought that *C. gattii* was restricted to tropical and subtropical regions [9], but the emergence of the outbreak events due to virulent strains in temperate areas of North America suggest a more global distribution of this yeast [5], [6]. In addition, independent cases have been reported from Mediterranean Europe [10].

The knowledge about the epidemiology of *C. gattii* is recent, although the first publications of meningeal cryptococcosis was in a child from the Congo in 1970 [11], while another proven infection occurred in a patient with a lumbar tumor as described by the French physician Ferdinand Curtis in 1896 [12].

*C. gattii* is a fungal pathogen globally set, with a potential primary ecological niche being associated in some way with decaying wood from a large range of tree species [13]. *C. gattii* has been associated with at least 54 species of trees native to tropical, subtropical and temperate regions [13], [14]. Currently, four major molecular types of *C. gattii* (VGI = AFLP4, VGII = AFLP6, VGIII = AFLP5 and VGIV = AFLP7) are accepted according to the characterization by PCR fingerprinting, Random Amplification of Polymorphic DNA (RAPD), Amplified Fragment Length Polymorphism (AFLP), MLMT and MLST analyses, which are different to the genotypes of *C. neoformans* (VNI to VNIV) [15]–[17]. In addition a number of hybrids, including inter-species (*C. neoformans* var. *neoformans* x *C. gattii* DB, *C. neoformans* var. *grubii* x *C. gattii* AB) and intra-species (*C. neoformans* var. *grubii* x *C. neoformans* var. *neoformans* AD) hybrids, have been identified [18], [19].

In Colombia, a national survey on cryptococcosis is ongoing since 1997, led by the Instituto Nacional de Salud in Bogotá (INS) and the Corporación para Investigaciones Biológicas in Medellin (CIB) [20], [21]. The objective of the present work was to analyze demographic, clinical and microbiological data concerning cryptococcosis caused by *C. gattii* received through the survey during the period of 1997–2011. Our overarching aim was to provide a broad brushstroke picture of this mycosis in Colombia.

## Materials and Methods

### Design

This is a descriptive study of the clinical, epidemiologic and microbiological characteristics of cases of cryptococcosis due to *C. gattii* in Colombia, identified through an ongoing national survey.

### Survey

The information was obtained by a survey designed according to the guidelines of the European Confederation of Medical Mycology, and processed by health professionals in different public and private health care institutions in Colombia, which also sent the corresponding strains from each case to the INS for centralized genotyping.

The survey contained the following information: year of diagnosis of cryptococcosis, patient's demographic data, such as gender and age, department (Colombia political divisions) and place of residence, risk factors for cryptococcosis (HIV infection, use of corticosteroids, autoimmune disease, organ transplants, presence of malignant solid tumors and hematologic malignancies, diabetes mellitus, liver cirrhosis, chronic kidney disease and sarcoidosis). In cases associated with HIV infection, we asked if cryptococcosis defined AIDS; the date of diagnosis of HIV, the clinical manifestations of cryptococcosis and the type of initial treatment. Also, diagnostic tests performed viz. direct examinations, cultures and the determination of the capsular antigen in serum and cerebrospinal fluid (CSF) and the findings of diagnostic imaging (radiographs of the chest and cross-sectional neuroimaging). In addition to the aforementioned information, the evolution of the disease was determined for patients from Norte de Santander.

### Ethical statement

The study was approved by the Ethics Committee of the CIB. Additionally, it had technical and ethical approval of the INS.

### Analysis

With the information received, a database was created using Biolomics ver. 7.5.44 (BioAware SA., Belgium), while the data were analyzed statistically using Epiinfo ver. 6.1 (CDC, USA).

### Case definition

A case was considered probable when there were clinical findings consistent with cryptococcosis. Cases were confirmed after isolation of a *Cryptococcus* spp from a normally sterile site, from sputum, bronchoalveolar lavage or biopsy.

### Incidence

The mean annual incidence rate was determined using as denominator the Colombian population census done in 2003, an intermediate year of the surveillance, determined by DANE [22]. For Norte de Santander, the population for the year 2003 was also used [22].

### Microbiology

Strain identification was done using conventional mycology techniques. Determination of species was performed by culturing the strains on CGB media [3]. In the first years of surveillance, serotype (B versus C) was determined using specific antisera available commercially (Iatron, Japan).

### Antifungal susceptibility

The susceptibility profiles to amphotericin B (AMB) using the E-test (BioMerieux, France) and to fluconazole (FCZ) and voriconazole (VCZ) using disc diffusion method M44-A described by the Clinical Laboratory Standards Institute (CLSI) were determined at the CIB laboratory. Quality control was done by including the *Candida albicans* strain ATCC 90028, which shows an inhibition range between 32–43 mm.

### Molecular type and mating type

Molecular type was determined in all strains using PCR fingerprinting with the primer (GTG)_5_ [15]. Mating type **a** or α (alpha) was determined using specific primers described previously [23].

### Multilocus Sequence Typing (MLST)

For 44 strains, seven unlinked genetic loci, including *CAP59, GPD1, LAC1, PLB1, SOD1, URA5* and the IGS1 region, were amplified following the ISHAM consensus MLST typing scheme for *C. neoformans* and *C. gattii* [16]. Amplification of loci was carried out in the Molecular Mycology Research Laboratory, University of Sydney at Westmead Hospital, Westmead, Australia, and the sequences were obtained commercially (Macrogen Inc., Korea). The generated sequences were manually edited using the Sequencher ver. 5.2 (Gene Codes Corporation, USA) software. With the concatenated sequences, a dendrogram showing the genetic relationships between the strains was constructed with the program Mega version 5.05 [24], based on maximum likelihood analysis. Allele types and sequence types (ST) were identified using the ISHAM consensus MLST database at mlst.mycologylab.org. The reference strains for *C. gattii* WM 179 (VGI = AFLP4; B/alpha), WM 178 (VGII = AFLP6; B/alpha), WM 175 (VGIII = AFLP5; B/alpha) and WM 779 (VGIV = AFLP7; C/alpha), were included in the genetic analyses for verification of the four major molecular types of *C. gattii* [15]. Genetic diversity was assessed by calculating the Simpson's diversity index (D) [25].

### Literature review

A search was performed using the Medline database about cryptococcosis by *C. gattii*, reported from January 1970 to 31 July 2014, its prevalence and the molecular types of clinical strains from the world using the following search terms in English: *Cryptococcus gattii*, epidemiology, meningitis, HIV, AIDS, children. Articles in Spanish and Portuguese were searched in the databases SciELO and Lilacs. Only available articles were included. In addition, some references cited in articles obtained in the primary search were included also.

## Results

### Prevalence, incidence of cases per year and place of origin

Over the period from January 1997 to December 2011, 1,209 surveys were completed, with corresponding cultured isolate, and were submitted from 76 centers in 25 departments of Colombia and Bogotá D.C. Of these, 45 (3.7%) corresponded to cases of cryptococcosis by *C. gattii*.

**Figure 1:**
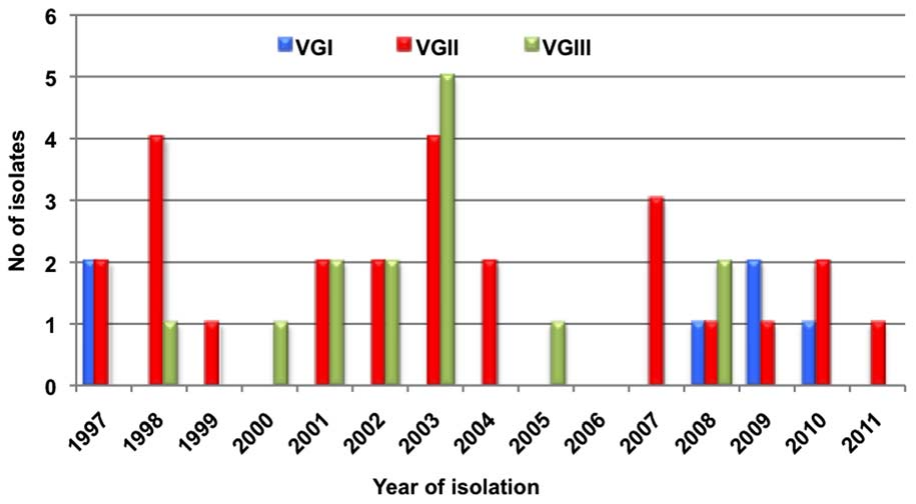
Distribution per year and molecular type of *Cryptococcus gattii* strains recovered in Colombia from 1997–2011.

**Figure 2:**
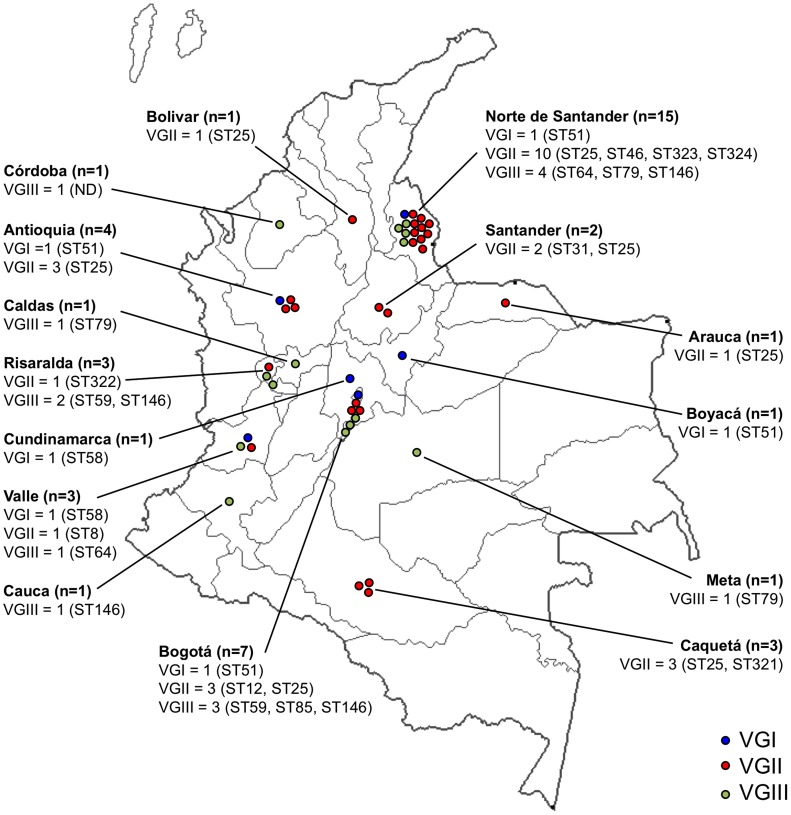
Distribution per department of the number, molecular type and STs of *Cryptococcus gattii* strains recovered in Colombia from 1997 to 2011. ND  =  ST was not determined.

The number of cases received per year is shown in Figure 1 and Table S1 and the origin and number of cases per department is shown in Figure 2 and Table S1. It should be noted that almost a third of the patients (33.3%) resided in Norte de Santander. The average annual incidence of *C. gattii* in Colombia was 0.07 cases per million inhabitants per year, but in Norte de Santander, it was 0.81 cases per million inhabitants per year.

### Demographic information

**Figure 3:**
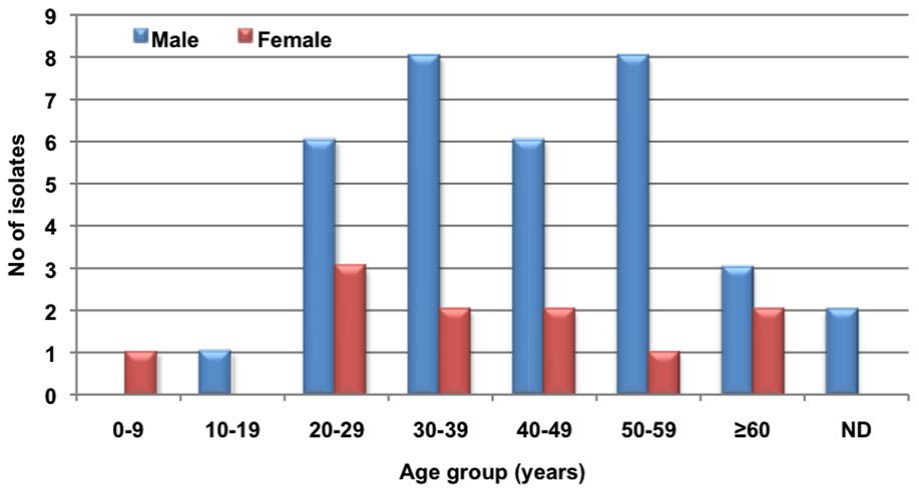
Distribution per age and gender of cryptococcosis cases reported in Colombia from 1997 to 2011.

A preponderance of males 34 (75.5%) was found, with the male: female ratio being 3.3∶1. The average age of the patients was 40±16 years with a range of4 to 68 years. The age and gender distribution is shown in Figure 3. There were 2 (4.5%) cases in children under 16 years-of-age.

### Risk factors

No predisposing risk factors were reported in 41 (91.1%) of patients. From the cases in which the risk factors were known, the most frequent were: HIV infection (3; 6.7%) and autoimmune disease with the use of steroids (1; 2.2%). Analysis of the Norte de Santander patients revealed that 93% had no apparent risk factor.

### Clinical findings

Clinical manifestations were available for 41 patients (Table 1). The most frequent abnormal physical findings were: headache (33; 80.5%), nausea and vomiting (23; 56.1%), mental changes (20; 48.8%) and visual alterations (18; 43.9%).

**Table 1:** Clinical manifestations of patients with *Cryptococcus gattii* cryptococcosis in Colombia, 1997–2011.

Signs and symptoms	n/Total	%
Headache	33/41	80.5
Nausea and vomiting	23/41	56.1
Mental alterations	20/41	48.8
Visual alterations	18/41	43.9
Fever	15/41	36.6
Meningeal signs	14/41	34.1
Intracranial hypertension with or without hydrocephalus	12/41	29.3
Seizures	7/41	17.1
Focal neurological signs	6/41	14.6
Cough	4/41	9.8

The most common clinical presentation was neurocryptococcosis (39 cases; 86.7%), followed by pulmonary cryptococcosis (3; 6.7%) and disseminated disease (2; 4.4%). Clinical presentation was not determined in 1 case (2.2%).

### Diagnostic imaging

In 21 (46.7%) cases results of chest X-ray were available, with 7 (33.3%). showing abnormalities. Computed tomography (CT) of the head was conducted in 15 (33.3%) cases with abnormalities reported in 10 (66.7%).

### Treatment

Initial antifungal therapy was reported in 30 (66.7%) surveys. AMB was used most often, in 29 (96.7%) patients.

### Microbiology

Various diagnostic methods were carried out for cryptococcal identification, including direct microscopy, determination of the capsular antigen in serum and CSF and culture of CSF and respiratory samples. The results of these tests are described in Table 2. The serotype was determined in 42 (93.3%) strains: 36 (85.7%) were serotype B, while 6 (14.3%) were serotype C.

**Table 2:** Clinical, microbiological and molecular data of *Cryptococcus gattii* cryptococcosis cases in Colombia, 1997–2011.

Type of exam	n/total	%
Positive direct examination	42/43	97.7
Capsular antigen detection	14/18	77.8
Serum reactive	6/8	75.0
CSF reactive	14/14	100.0
Positive culture	45/45	100.0
Origin of strains		
CSF	40/45	88.9
CSF and blood	2/45	4.4
Bronchoalveolar lavage	1/45	2.2
Lung biopsy	1/45	2.2
Sputum	1/45	2.2
Serotype		
B	36/42	85.7
C	6/42	14.3
Molecular type		
VGI	6/45	13.3
VGII	25/45	55.6
VGIII	14/45	31.1
Mating type		
a	21/45	46.7
alpha	24/45	53.3

### Antifungal susceptibility

Susceptibility to antifungals was determined for 42/45 *C. gattii* strains. All strains were susceptible to AMB (MIC <2 µg/ml). Thirteen (31%) were susceptible (MIC ≤8 µg/ml), 14 (33.3%) susceptible dose-dependent (SDD) (MIC 16–32 µg/ml) and 15 (35.7%) resistant (MIC ≥64 µg/ml) to FCZ. With regard to VCZ, 39 (92.9%) were susceptible (MIC ≤1 µg/ml), 1 (2.4%) SDD (MIC 2 µg/ml) and 2 (4.8%) resistant (MIC ≥4 µg/ml).

### Molecular type and mating type

The molecular types of the 45 VG isolates in order of frequency were: VGII 25 (55.6%), VGIII 14 (31.1%), and VGI 6 (13.3%) (Table 2). The distribution of isolates per molecular type per department is shown in Figure 2. In regard to mating type of the strains, 24 (53.3%) were mating type α and 21 (46.7%) were mating type **a** (Table 2).

#### Multilocus sequence typing (MLST)

**Figure 4:**
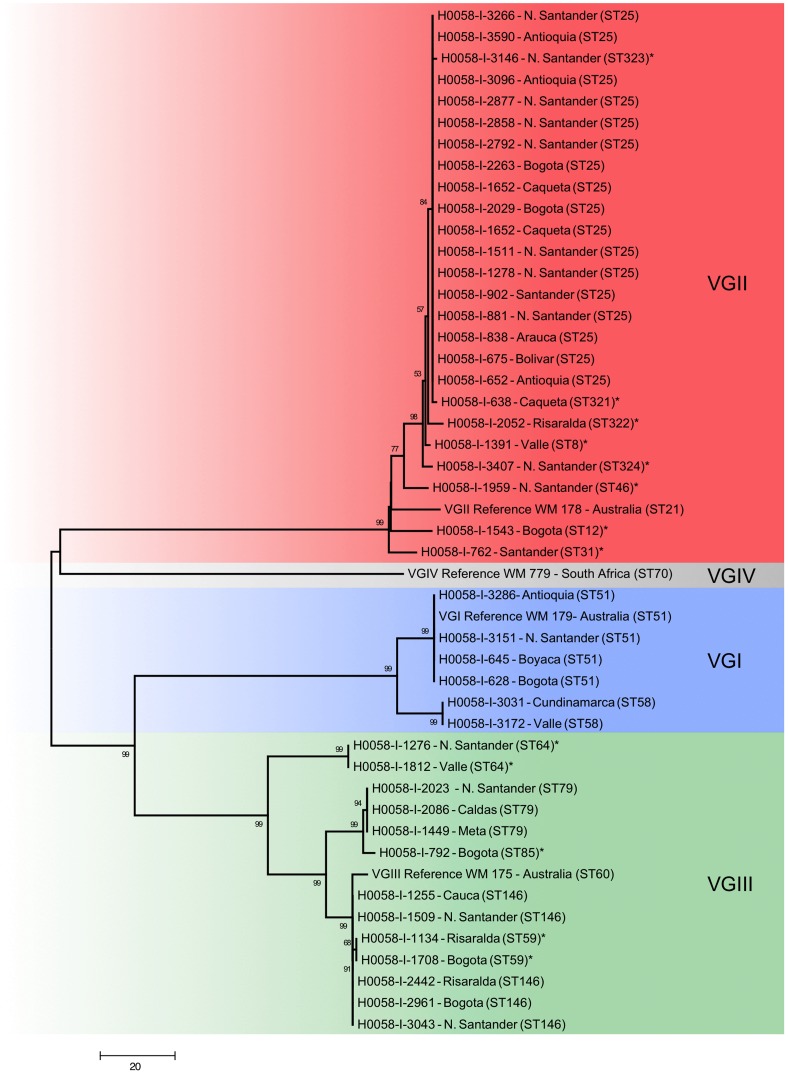
Genetic relationship of the *Cryptococcus gattii* strains causing cryptococcosis in Colombia from 1997 to 2011. Sequence types identified for the first time in this study are indicated with an asterisk (*).

Among the 44 isolates genotyped, 16 sequence types (ST) were identified: two STs amongst the VGI strains, nine STs amongst the VGII strains (with ST25 being the most common; 17 (68%) strains) and five STs amongst the VGIII strains (Table 3). Eight STs of the VGII strains (ST8, ST12, ST31, ST46, ST321, ST322, ST323 and ST324) and three of the VGIII strains (ST59, ST64 and ST85) were identified for the first time in this study. The genetic relationship of the studied strains is shown in Figure 4, while the distribution of the STs per department is shown in Figure 2. The MLST data was incorporated into the *C. neoformans* and *C. gattii* MLST databases accessible at http://mlst.mycologylab.org and the sequences of identified alleles were deposited in GenBank (Table S2).

**Table 3:** Molecular type, mating type, allele types and sequence types of Colombian *Cryptococcus gattii* clinical strains.

Molecular type RFLP/AFLP	Strain number	Other collection number	State	Mating type	*CAP59*	*GPD1*	IGS1	*LAC1*	*PLB1*	*SOD1*	*URA5*	ST
VGI/AFLP4	H0058-I-628	WM 2039	Bogotá	a	16	5	3	5	5	32	12	51
	H0058-I-645		Boyacá	alpha	16	5	3	5	5	32	12	51
	H0058-I-3151		Norte de Santander	alpha	16	5	3	5	5	32	12	51
	H0058-I-3286		Antioquia	alpha	16	5	3	5	5	32	12	51
	H0058-I-3031		Cundinamarca	alpha	16	11	13	19	15	34	14	58
	H0058-I-3172		Valle	alpha	16	11	13	19	15	34	14	58
VGII/AFLP6	H0058-I-652	WM 2043	Antioquia	a	2	6	25	4	18	12	10	25
	H0058-I-675	WM 04.77, LA584	Bolívar	alpha	2	6	25	4	18	12	10	25
	H0058-I-838	WM 2065, LA596	Arauca	a	2	6	25	4	18	12	10	25
	H0058-I-881	WM 08.295, LA225	Norte de Santander	a	2	6	25	4	18	12	10	25
	H0058-I-902	WM 2069, LA 603	Santander	alpha	2	6	25	4	18	12	10	25
	H0058-I-1278	WM 05.275	Norte de Santander	a	2	6	25	4	18	12	10	25
	H0058-I-1511	WM 05.399	Norte de Santander	a	2	6	25	4	18	12	10	25
	H0058-I-1652		Caquetá	a	2	6	25	4	18	12	10	25
	H0058-I-2029	WM 05.394	Bogotá	a	2	6	25	4	18	12	10	25
	H0058-I-2151		Caquetá	a	2	6	25	4	18	12	10	25
	H0058-I-2263		Bogotá	alpha	2	6	25	4	18	12	10	25
	H0058-I-2792	WM 08.297	Norte de Santander	a	2	6	25	4	18	12	10	25
	H0058-I-2858	WM 08.298	Norte de Santander	a	2	6	25	4	18	12	10	25
	H0058-I-2877	WM 08.299	Norte de Santander	a	2	6	25	4	18	12	10	25
	H0058-I-3096	WM 11.119	Antioquia	alpha	2	6	25	4	18	12	10	25
	H0058-I-3266		Norte de Santander	a	2	6	25	4	18	12	10	25
	H0058-I-3590		Antioquia	a	2	6	25	4	18	12	10	25
	H0058-I-1391	WM 05.349	Valle	a	2	6	10	4	18	12	2	8*
	H0058-I-638	WM 04.76, LA 221	Caquetá	alpha	2	6	45*	4	18	12	10	321*
	H0058-I-3146		Norte de Santander	a	2	6	95*	4	18	12	10	323*
	H0058-I-3407		Norte de Santander	a	2	21	25	4	41*	12	2	324*
	H0058-I-1543	WM 05.397	Bogotá	alpha	11*	21	6	4	16	5*	2	12*
	H0058-I-2052		Risaralda	a	14	21	94*	4	18	107*	7	322*
	H0058-I-762	WM 04.78, LA224	Santander	alpha	4	6	6	4	18	22*	2	31*
	H0058-I-1959	WM 05.272	Norte de Santander	a	26*	6	4	28	2	27*	2	46*
VGIII/AFLP5	H0058-I-792	WM 2063, LA594	Bogotá	alpha	29	9	5	9	4	28	21	85*
	H0058-I-1442	WM 11.102	Meta	alpha	29	7	5	2	4	28	21	79
	H0058-I-2023	WM 11.105	Norte de Santander	alpha	29	7	5	2	4	28	21	79
	H0058-I-2086	WM 11.106	Caldas	alpha	29	7	5	2	4	28	21	79
	H0058-I-1255		Cauca	alpha	18	3	1	3	17	28	19	146
	H0058-I-1509		Norte de Santander	alpha	18	3	1	3	17	28	19	146
	H0058-I-2442	WM 11.112	Risaralda	alpha	18	3	1	3	17	28	19	146
	H0058-I-2961	WM 11.118	Bogotá	alpha	18	3	1	3	17	28	19	146
	H0058-I-3043		Norte de Santander	alpha	18	3	1	3	17	28	19	146
	H0058-I-1134	WM 2088	Risaralda	a	18	3	1	3	17	38	19	59*
	H0058-I-1708	WM 11.104	Bogotá	a	18	3	1	3	17	38	19	59*
	H0058-I-1276	WM 2114, LA705	Norte de Santander	alpha	43	31	63*	43	32	39	18	64*
	H0058-I-1812	WM 05.371	Valle	alpha	43	31	63*	43	32	39	18	64*

Note: *New allele or sequence type.

To estimate the genetic diversity of the isolates studied, the Simpson's Diversity index (*D*) was calculated. In general, *C. gattii* strains from Colombia were highly diverse (*D* = 0.20). The greatest diversity was identified in the molecular type VGIII (*D* = 0.26), followed by VGII (*D* = 0.48) and VGI (*D* = 0.56).

### Follow-up

For 11 patients treated in Norte de Santander, follow-up was undertaken. Nine (81.8%) were discharged alive, while 2 (18.2%) died during hospitalization.

### Literature review

Table S3 reflects the prevalence of cryptococcosis by *C. gattii* around the world, as well as their molecular types, when reported [26]–[112].

## Discussion

The incidence of cryptococcosis caused by *C. gattii* globally is generally low overall [113]. The mean annual incidence found in this study for Colombia, of 0.07 cases per million inhabitants per year reflects this rarity. Similar findings (0.09 cases per million per year) have been described from New Zealand [111]. However, some regions and population groups of the world have very high incidence, such as Papua New Guinea (43 cases per million per year) [114], the Australian aborigines domiciled in the Northern Territory (6.3 cases per million per year) [115] and British Columbia, Canada (5.8 cases per million per year) [62]. It is striking that the incidence of this disease in the department Norte de Santander is eleven times higher than the national (0.81 cases per million per year), which puts it at the level described for Australia as a continent(0.61 cases per million per year) [115].

Regarding the prevalence of *C. gattii* infections, the value of 3.7% found in this survey reflects Colombia is a country of overall low prevalence, despite being in the torrid zone of the planet. However, Norte de Santander has a high prevalence of 33.3% (60% in patients without AIDS) [59], placing this region of Colombia on level with other countries of high prevalence such as Papua New Guinea [107], [108], the Northern Territory of Australia [110], and Brazil [27] (Table S3). In Brazil, there is a clear difference between the North and South. In the area that covers the North and Northeast regions, the prevalence is very high, with values above 20% [28]–[33], while in the regions Center West, Southeast and South, the prevalence is low [34]–[48]. Interestingly, a high proportion of cases of cryptococcosis due to *C. gattii* in the northern region of Brazil have been present in HIV-negative children [28]. A high prevalence of disease has also been reported from Venezuela [15], [52]–[54], French Guiana [55] Vietnam [100] and Hong Kong (China) [87]. Similar findings have been reported in some African countries, especially Botswana and Malawi, where a prevalence of 13% of *C. gattii* cryptococcosis was found in patients with AIDS, a high value for this type of population [76]. Another study reported a prevalence of 30% of *C. gattii* in hospitalized AIDS patients in Botswana [77] (Table S3). On the other hand, the overall prevalence in Europe is low, and many of the cases are from people immigrating coming from other regions in the world where cryptococcosis is more common[10], [68], [69]. Against this trend, an apparently endemic strain from an environmental source in the Netherlands was recently reported [116]. A low prevalence of cryptococcosis by *C. gattii* was also reported from South Africa, where AIDS-related cryptococcosis due to *C. neoformans* is epidemic [82]. Similar findings have been reported from other countries of the African continent [79]–[81], [84], in Mexico [50], [51], Argentina [15], [56], [57] and Asia including China (without Hong Kong) [85], [86], India [93]–[95] and in Southeast Asia [96]–[99] but excluding Vietnam and Thailand (Table S3).

As in cryptococcosis patients infected with *C. neoformans*, disease referable to *C. gattii* is more frequent in men [117], typically young adults [115], although in this study we found children also and older patients.

The presence of cryptococcosis by *C. gattii* in children domiciled in tropical areas in Brazil [28], [34], French Guiana [55], and parts of Australia (Meyer, unpublished) in a remarkable finding that invites further enquiry and research.

The majority (91.1%) of the Colombian patients described in the current study did not report any apparent risk factor. There were, however, some cases in patients with AIDS or immunosuppressed by another means. The behavior of Colombian patients is in accord with the descriptions of previous studies [111], [118], [119]. The epidemiology of cryptococcosis by *C. gattii* has changed in the last decade, with the recognition that disease affects immunocompetent and immunosuppressed patients, including patients with AIDS, in regions of the world outside of the tropical and subtropical areas [14], [120], [121]. In Australia, where this trend is also observed, the number of immunosuppressed patients without AIDS increased from 9% to 28% [115]. In British Columbia, Canada the risk factors for infection with *C. gattii* were the use of oral steroids, the presence of pneumonia and other lung diseases [120]. Also, cryptococcosis was more frequent in patients older than 50 years, active smokers, HIV-positive individuals and a history of invasive cancer [120]. In the Pacific Northwest of the United States, patients infected with the outbreak strains of *C. gattii*, when compared with patients infected with different strains, were significantly more likely to have predisposing conditions and respiratory symptoms and a lower probability of having CNS involvement [121]. In Colombian patients, the most common clinical presentation was the CNS involvement with predominance of cryptococcal meningitis and intracranial hypertension. These clinical manifestations have been described in Latin America [39], Asia [96], Africa [82], [83], Australia [115] and Papua New Guinea [107], [108]. This contrasts with the patients described in Vancouver, the Pacific Northwest of the United States and the Northern Territory of Australia where primary lung disease with granuloma formation predominates [120], [121].

It is believed that *C. gattii* is clinically more virulent than *C. neoformans*, as well as being more likely to be a primary pathogen, with a propensity to cause multiple cryptococcal granulomas in the lungs and the brain of affected patients [111], [118]. In a mouse model, clinical isolates of *C. gattii* from the outbreak in British Columbia induced an inflammatory response less protective because the organisms somehow inhibit the migration of neutrophils towards the sites of infection. Additionally, in contrast to *C. neoformans*, they fail to induce the production of protective cytokines [122]. *In vitro* studies of the same isolates showed that dendritic cells are able to destroy cryptococci, but *C. gattii* evades adaptive immunity by preventing maturation of those cells and causing an inadequate activation and proliferation of T cells [123]. Recently, it has been shown in mice that infection by *C. gattii* decreases the effective response of Th1/Th17 mediated by dendritic cells and regulates the low expression of lung cytokines, resulting in an inability to produce a protective immunity in immunocompetent hosts [124].

Compared to patient with *C neoformans* infections, meningoencephalitis caused by *C. gattii* responds more slowly to antifungal therapy, and patients require a longer duration of treatment [111], [119]. Diagnostic imaging of the lung and brain are typically abnormal in patients infected with *C. gattii*. In the current study a third of the patients presented with gross lung abnormalities.

In immunocompetent patients affected by *C. gattii*, pulmonary nodules or masses with diameters ranging from 5 to 52 mm in diameter and focal areas of consolidation have been described [125]. Some clinical considerations in the differentiation of the infections caused by *C. gattii* and *C. neoformans* were recently revised [126].

The diagnosis of the vast majority of the Colombian patients reported in the study was obtained by analysis of CSF samples. Direct microscopic examination showed high positivity (97.7%), compared with much lower rates reported for this test in patients without AIDS [87], [127]. Also, capsular antigen in the CSF was reactive in all cases as measured by latex agglutination. Definitive diagnosis was established in all patients using culture, and the use of CBG agar established which cases were caused by *C. gattii*. The most frequently identified serotype in Colombian clinical strains was serotype B (85.7%), which is the most prevalent serotype in clinical and environmental samples. *C. gattii* serotype C was less common although has been associated with AIDS and with immunocompetent patients [13].

In Colombian patients, three of the four molecular types of *C. gattii* were found with a clear predominance of VGII, followed by VGIII and to a lesser extent VGI. The preponderance of the molecular type VGII in Colombia is similar to that reported in Western Australia [109], [112], Brazil (especially in the Northern region) [27] and Venezuela [15]. Equally, the molecular type VGII is the main biotype responsible for outbreaks in British Columbia, Canada [63] and the Pacific Northwest of the United States [64]. Hence the importance of determining the taxonomy of *C. gattii* strains because of the epidemic potential associated with the VGII molecular type.

The emergence of specific genotypes of endemic and epidemic disease has been reflected in the large global effort being made to increase knowledge about the population genetics of *C. neoformans* and *C. gattii* [15], [112]. MLST data has demonstrated that Colombian *C. gattii* strains are genetically diverse. In spite of the small number of isolates studied, several genotypes were identified belonging to the full range of molecular types, in contrast to the less diverse and rather clonal *C. gattii* populations reported in other countries such as Canada, USA, eastern Australia and Thailand, where few genotypes have been identified amongst a much larger number of strains [5], [6], [23], [63], [99]. The identification of the same STs in different departments (Figure 2), for example the prevalent VGII ST25 found in 7 different departments, the VGI ST51 and ST58, and the VGIII ST64, ST79 and ST146, suggests the circulation of genotypes in the country.

The finding of STs previously reported in other parts of the world, shows a wider geographic dispersion of some *C. gattii* genotypes, especially amongst the VGI isolates, for which the herein identified STs have been reported from several countries; ST51 was previously found in Australia, China, India, Mexico, Papua New Guinea and USA [10], [16], [128], while ST58 had already been described from the Netherlands, Germany and USA [68]. Among the VGII isolates, the commonest ST identified in this study, ST25, was already reported in one isolate from Aruba [129], while the STs among the VGIII isolates, ST79 and ST146, were reported in two and one isolates from Mexico and the USA, respectively [129].

In Colombia, the majority of the strains of *C. gattii* are mating type **a** [130], in contradistinction to what was found in British Columbia, Canada [5] and of the Pacific coast of the United States [6], and southwestern Western Australia where the main mating type is α, which could suggest the possibility of genetic exchange that could have had an impact on the origin of the outbreaks. *C. gattii* has been recovered frequently from the environment in Colombia and in the city of Cúcuta (Norte de Santander), with both serotypes B and C having been cultured [131], [132].

The initial treatment of cryptococcosis in almost all patients from Colombian was done with AMB, with or without FCZ, which is in agreement with the results of susceptibility testing of all strains of *C. gattii* to these antifungals and according with the international guidelines for countries with limited resources [133]. The resistance of approx. half of the Colombian strains to fluconazole is a phenomenon that is being studied by the group of the CIB (C de Bedout, personal communication).

## Supporting Information

Checklist S1 STROBE checklist.
(DOCX)

Table S1 Number of *Cryptococcus gattii* cryptococcosis cases in Colombia, per state, year and molecular type. Number of cases in brackets.
(DOCX)

Table S2 Gene Bank accession numbers for the allele types of Colombian *Cryptococcus gattii* clinical strains.
(DOCX)

Table S3 *Cryptococcus gattii*: Prevalence and molecular type of clinical isolates reported worldwide.
(DOCX)
