# Supplementary material for: Retrospective Study of the Epidemiology and Clinical Manifestations of Cryptococcus gattii Infections in Colombia from 1997–2011
Source: PLoS Negl Trop Dis. 2014 Nov 20;8(11):e3272. doi: 10.1371/journal.pntd.0003272 (PMC4238989; doi:10.1371/journal.pntd.0003272)
Supplement: Table S1 — Number of Cryptococcus gattii cryptococcosis cases in Colombia, per state, year and molecular type. Number of cases in brackets. (DOCX) [file pntd.0003272.s002.docx]

Supplementary Tables

Table S1: Number of *Cryptococcus gattii* cryptococcosis cases in Colombia, per state, year and molecular type. Number of cases in brackets.

| Department | 1997 | 1998 | 1999 | 2000 | 2001 | 2002 | 2003 | 2004 | 2005 | 2007 | 2008 | 2009 | 2010 | 2011 | Total |
| --- | --- | --- | --- | --- | --- | --- | --- | --- | --- | --- | --- | --- | --- | --- | --- |
| Antioquia |  | VGII (1) |  |  |  |  |  |  |  |  | VGII (1) |  | VGI (1) | VGII (1) | **4** |
| Arauca |  | VGII (1) |  |  |  |  |  |  |  |  |  |  |  |  | **1** |
| Bogotá | VGI (1) | VGIII (1) |  |  |  | VGII (1) | VGII (1)  VGIII (1) | VGII (1) |  |  | VGIII  (1) |  |  |  | **6** |
| Bolívar | VGII (1) |  |  |  |  |  |  |  |  |  |  |  |  |  | **1** |
| Boyacá | VGI (1) |  |  |  |  |  |  |  |  |  |  |  |  |  | **1** |
| Caldas |  |  |  |  |  |  | VGIII (1) |  |  |  |  |  |  |  | **1** |
| Caquetá | VGII (1) |  |  |  |  |  | VGII (1) | VGII (1) |  |  |  |  |  |  | **3** |
| Cauca |  |  |  |  | VGIII (1) |  |  |  |  |  |  |  |  |  | **1** |
| Córdoba |  |  |  |  |  |  | VGIII (1) |  |  |  |  |  |  |  | **1** |
| Cundinamarca |  |  |  |  |  |  |  |  |  |  | VGI  (1) |  |  |  | **1** |
| Meta |  |  |  |  |  | VGIII (1) |  |  |  |  |  |  |  |  | **1** |
| Norte de Santander |  | VGII (1) |  |  | VGII (1)  VGIII (1) | VGII (1)  VGIII (1) | VGII (1)  VGIII (1) |  |  | VGII (3) | VGIII (1) | VGI (1)  VGII (1) | VGII (2) |  | **15** |
| Risaralda |  |  |  | VGIII (1) |  |  | VGII (1) |  | VGIII (1) |  |  |  |  |  | **2** |
| Santander |  | VGII (1) | VGII (1) |  |  |  |  |  |  |  |  |  |  |  | **2** |
| Valle |  |  |  |  | VGII (1) |  | VGIII (1) |  |  |  |  | VGI (1) |  |  | **3** |
| Total | 4 | 5 | 1 | 1 | 4 | 4 | 9 | 2 | 1 | 3 | 4 | 3 | 3 | 1 | 45 |
