# Supplementary material for: Retrospective Study of the Epidemiology and Clinical Manifestations of Cryptococcus gattii Infections in Colombia from 1997–2011
Source: PLoS Negl Trop Dis. 2014 Nov 20;8(11):e3272. doi: 10.1371/journal.pntd.0003272 (PMC4238989; doi:10.1371/journal.pntd.0003272)
Supplement: Table S2 — Gene Bank accession numbers for the allele types of Colombian Cryptococcus gattii clinical strains. (DOCX) [file pntd.0003272.s003.docx]

Table S2. Gene Bank accession numbers for the allele types of Colombian *Cryptococcus gattii* clinical strains

| Loci | Allele | GenBank accession No. |
| --- | --- | --- |
| *CAP59* | CAP59 allele 2 | KF756945 |
|  | CAP59 allele 4 | KF756946 |
|  | CAP59 allele 11 | KF756947 |
|  | CAP59 allele14 | KF756948 |
|  | CAP59 allele 16 | KF756949 |
|  | CAP59 allele 18 | KF756950 |
|  | CAP59 allele 26 | KF756951 |
|  | CAP59 allele 29 | KF756952 |
|  | CAP59 allele 43 | KF756953 |
| *GPD1* | GPD1 allele 3 | KF756954 |
|  | GPD1 allele 5 | KF756955 |
|  | GPD1 allele 6 | KF756956 |
|  | GPD1 allele 7 | KF756957 |
|  | GPD1 allele 9 | KF756958 |
|  | GPD1 allele 11 | KF756959 |
|  | GPD1 allele 21 | KF756960 |
|  | GPD1 allele 31 | KF756961 |
| IGS1 | IGS1 allele 1 | KF756962 |
|  | IGS1 allele 3 | KF756963 |
|  | IGS1 allele 4 | KF756964 |
|  | IGS1 allele 5 | KF756965 |
|  | IGS1 allele 6 | KF756966 |
|  | IGS1 allele 10 | KF756967 |
|  | IGS1 allele 13 | KF756968 |
|  | IGS1 allele 25 | KF756969 |
|  | IGS1 allele 45 | KF756970 |
|  | IGS1 allele 63 | KF756971 |
|  | IGS1 allele 94 | KF756972 |
|  | IGS1 allele 95 | KF756973 |
| *LAC1* | LAC1 allele 2 | KF756974 |
|  | LAC1 allele 3 | KF756975 |
|  | LAC1 allele 4 | KF756976 |
|  | LAC1 allele 5 | KF756977 |
|  | LAC1 allele 9 | KF756978 |
|  | LAC1 allele 19 | KF756979 |
|  | LAC1 allele 28 | KF756980 |
|  | LAC1 allele 43 | KF756981 |
| *PLB1* | PLB1 allele 2 | KF756982 |
|  | PLB1 allele 4 | KF756983 |
|  | PLB1 allele 5 | KF756984 |
|  | PLB1 allele 15 | KF756985 |
|  | PLB1 allele 16 | KF756986 |
|  | PLB1 allele 17 | KF756987 |
|  | PLB1 allele 18 | KF756988 |
|  | PLB1 allele 32 | KF756989 |
|  | PLB1 allele 41 | KF756990 |
| *SOD1* | SOD1 allele 5 | KF756991 |
|  | SOD1 allele 12 | KF756992 |
|  | SOD1 allele 22 | KF756993 |
|  | SOD1 allele 27 | KF756994 |
|  | SOD1 allele 28 | KF756995 |
|  | SOD1 allele 32 | KF756996 |
|  | SOD1 allele 34 | KF756997 |
|  | SOD1 allele 38 | KF756998 |
|  | SOD1 allele 39 | KF756999 |
|  | SOD1 allele 107 | KF757000 |
| *URA5* | URA5 allele 2 | KF757001 |
|  | URA5 allele 7 | KF757002 |
|  | URA5 allele 10 | KF757003 |
|  | URA5 allele 12 | KF757004 |
|  | URA5 allele 14 | KF757005 |
|  | URA5 allele 18 | KF757006 |
|  | URA5 allele 19 | KF757007 |
|  | URA5 allele 21 | KF757008 |
