# Supplementary material for: Retrospective Study of the Epidemiology and Clinical Manifestations of Cryptococcus gattii Infections in Colombia from 1997–2011
Source: PLoS Negl Trop Dis. 2014 Nov 20;8(11):e3272. doi: 10.1371/journal.pntd.0003272 (PMC4238989; doi:10.1371/journal.pntd.0003272)
Supplement: Table S3 — Cryptococcus gattii: Prevalence and molecular type of clinical isolates reported worldwide. (DOCX) [file pntd.0003272.s004.docx]

**Table S3.** *Cryptococcus gattii*: Prevalence and molecular type of clinical isolates reported worldwide

| **Latin America and Caribbean** | | |
| --- | --- | --- |
| **Country** | **Prevalence (%)** | **Molecular types** |
| **Brazil** | | |
| National | | |
| [Rozenbaum](http://www.ncbi.nlm.nih.gov/pubmed?term=Rozenbaum%20R%5BAuthor%5D&cauthor=true&cauthor_uid=1435954), 1994 [26] | 9.6 | ND |
| Trilles, 2008 [27] | 25.1 | VGI, VGII, VGIII |
| North | | |
| Correa, 1999 [28]. Pará (Children) | 64.3 | ND |
| Santos, 2008 [29]. Pará | 49.9 | VGI, VGII |
| Freire, 2012 [30]. Amazonas | 29.8 | VGI, VGII |
| Da Silva, 2012 [31]. Amazonas | 22.5 | VGII |
| Northeast | | |
| Martins, 2011 [32]. Piauí | 38.1 | VGII |
| Matos, 2012 [33]. Salvador | 21.0 | VGII |
| Central-West | | |
| Tsujisaki, 2013 [34] (Cryptococcemia) | 2.1 | VGII |
| Souza, 2005 [35]. Goiás | 5.7 | ND |
| Souza, 2010 [36]. Goiás | 3.2 | VGII |
| Hasimoto e Souza, 2013 [37]. Goiás | 5.6 | VGII |
| Favalessa, 2014 [38]. Mato Grosso (one case) | ND | VGII |
| Lindenberg, 2008 [39]. Mato Grosso do Sul | 10.4 | ND |
| Favalessa, 2009 [40]. Mato Grosso do Sul | 16.2 | ND |
| Southeast | | |
| Ohkusu, 2002 [41]. Sao Paulo (53,3% HIV negative) | 10.7 | ND |
| Almeida, 2007 [42]. Sao Paulo | 4.8 | ND |
| Motta, 2010 [43]. Sao Paulo | 23 | ND |
| Nascimiento 2014 [44]. Sao Paulo (case) | ND | VGI |
| Moreira, 2006 [45]. Minas Gerais | 7.3 | ND |
| Silva, 2008 [46]. Minas Gerais | 11.7 | ND |
| Mora, 2012 [47]. Minas Gerais | 4.9 | ND |
| South | | |
| Casali, 2003 [48]. Rio Grande do Sul | 10.5 | VGIII |
| **Mexico** | | |
| López-Martínez, 1996 [49] | 30.0 | ND |
| Castañón-Olivares, 2000 [50] | 10.4 | ND |
| Castañón-Olivares, 2009 [51] | 11.1 | VGI, VGII, VGIII,VGIV |
| **Venezuela** | | |
| Villanueva, 1989 [52] | 29.6 | ND |
| Meyer, 2003 [15] | 20.0 | VGII, VGIII |
| Pérez, 2008 [53] | 9.1 | ND |
| Pérez, 2009 [ 54] | 11.8 | ND |
| **French Guiana** |  |  |
| Debourgogne, 2011 [55] | 22.7 | ND |
| **Argentina** | | |
| Bava, 1992 [56] | 3.8 | ND |
| Bava, 1997 [57] | 4.4 | ND |
| Meyer, 2003 [15] | 3.8 | VGII, VGIII |
| **Peru** | | |
| Bustamante, 1998 [58]  Meyer, 2003 [15] | 2.9  ND | ND  VGI |
| **Colombia** | | |
| Lizarazo, 2012 [59].  Norte de Santander (60% HIV negative) | 22.2 | VGI, VGII, VGIII |
| Lizarazo, 2013 [This study] | 3.7 | VGI, VGII, VGIII |
| **Other countries** | | |
| **Guatemala:** Meyer, 2003 [15] | ND | VGIII |
| **Paraguay**: Kwon Chung, 1984 [9] | ND | ND |
| **Argentina:** Cattana, 2013 [60].  El Chaco Province | 3.8 | VGI |
| **Cuba**: Illnait-Zaragozí MT, 2013 [61]  (one case) | ND | VGIII |

| **North America** | | |
| --- | --- | --- |
| **Country** | **Prevalence (%)** | **Molecular types** |
| **Canada** | | |
| Vancouver region | | |
| Hoang, 2004 [62] (Before outbreak) | 5.0 | ND |
| Galanis, 2010 [63] | Outbreak | VGIIa, VGIIb |
| **United States** | | |
| Lockhart, 2013 [64]. Pacific Northwest | Outbreak | VGIIa, VGIIb, VGIIc |
| Outside the Pacific Northwest | ND | VGI, VGIII |
| Pacific Northwest | | |
| Byrnes, 2010 [65] | Outbreak | VGIIa, VGIIb, VGIIc |
| Southern California | | |
| Byrnes, 2011 [66] (AIDS patients) | ND | VGIII |
| Chaturvedi, 2005 [67] (AIDS patients) | 12.3 | ND |

| **Europa** | | |
| --- | --- | --- |
| **Country** | **Prevalence (%)** | **Molecular types** |
| Tintelnot, 2004 [68]. Germany, Austria and Switzerland | 4.0 | VGI, VGIII, VGIV |
| Viviani, 2006 [69]. Austria, Germany, Greece, France and United Kingdom | 1.1 | ND |
| Hagen 2012 [10]. Europe | ND | VGI, VGII,VGIII, VGIV |
| **France** | | |
| Dromer, 1996 [70] | 0.3 | ND |
| **Portugal** | | |
| Maduro, 2012 [71] | 0.8 | VGII |
| **Other countries** (one case each) | | |
| Greece: Velegraki, 2001 [72] | ND | ND |
| Spain: Colom, 2004 [73] | ND | ND |
| The Netherlands: Hagen, 2010 [74] | ND | ND |
| Italy: Iatta, 2012 [75] | ND | ND |

| **Africa** | | |
| --- | --- | --- |
| **Country** | **Prevalence (%)** | **Molecular types** |
| **Botswana** | | |
| Litvintseva, 2005 [76] (AIDS patients) | 13.7 | VGIV |
| Steele, 2010 [77] (AIDS patients) | 30.0 | ND |
| **Democratic Republic of the Congo** | | |
| Swinne, 1986 [78]   - (1951-1969) (pre-AIDS) - (1970-1985) (AIDS) | 85.7  0 | ND  ND |
| **Kenya** | | |
| Bii, 2007 [79] | 2.5 | ND |
| Mdodo, 2011 [80] | 6.0 | ND |
| **Malawi** | | |
| Litvintseva, 2005 [76] (AIDS patients) | 13.3 | VGIV |
| **Rwanda** | | |
| Bogaerts, 1999 [81] (AIDS patients) | 1.6 | ND |
| **South Africa** | | |
| Morgan, 2006 [82] | 2.4 | ND |
| Meiring, 2012 [83]   - < 15 years old - ≥15 years old | 3.0  7.0  3.0 | ND  ND  ND |
| **Zimbabwe** | | |
| Heyderman, 1998 [84] | 2.2 | ND |

| **Asia** | | | |
| --- | --- | --- | --- |
| **Country** | **Prevalence (%)** | **Molecular types** | |
| **China** | | | |
| Chen, 2008 [85] | 7.0 | VGI | |
| Southeast, Shangai | | | |
| Li, 2012 [86] | 14.8 | ND | |
| **Hong Kong** | | | |
| Lui, 2006 [87] | 21.4 | VGI, VGII | |
| **Taiwan** | | | |
| Liaw, 2010 [88] | 1.0 | VGI | |
| Tseng, 2013 [89] | 4.1 | VGI, VGII | |
| **Korea** | | | |
| Choi, 2010 [90] | 4.0 | VGII, VGIII | |
| Hwang, 2012 [91] | 2.4 | VGI, VGII, VGIII | |
| **India** | | | |
| Padhye, 1993 [92] | 16.7 | ND | |
| Banerjee, 2004 [93] | 8.6 | ND | |
| Jain, 2005 [94] | 8.8 | ND | |
| Nagarathna, 2010 [95] | 2.8 | ND | |
| **Malaysia** | | | |
| Tay, 2010 [96] | 11.5 | VGI, VGII | |
| **Singapore** | | | |
| Chan, 2014 [97] | 3.9 | VGII | |
| **Thailand** | | | |
| Poonwan, 1997 [98] | 4.3 | ND | |
| Kaocharoen, 2013 [99] | 3.4 | VGII, VGI | |
| **Vietnam** | | | |
| Chau, 2010 [100] | 30.0 | VGI, VGIII | |
| Day, 2011 [101] | 27.4 | VGI, VGII | |
| **Other countries** (one case each) | | | |
| **Cambodia:** Kwon Chung, 1984 [9] |  | |  |
| **Japan:** Tsunemi, 2001 [102]  Okamoto, 2010 [103] | ND  ND | | ND  VGIIa |
| **Nepal:** Kwon Chung, 1984 [9] | ND | | ND |
| **Singapore:** Taylor, 2002 [104]  Koh, 2002 [105]  Lingegowda, 2011 [106] | ND  ND  ND | | ND  ND  ND |

| **Oceania** | | |
| --- | --- | --- |
| **Country** | **Prevalence (%)** | **Molecular types** |
| **Papua, New Guinea** | | |
| Laurenson, 1996 [107] | 63.6 | ND |
| Seaton, 1997 [108] | 95.0 | ND |
| Campbell, 2005 [109] | ND | VGI, VGII, VGIII |
| **Australia** | | |
| National | | |
| Ellis, 1987 [110] | 50.0 | ND |
| Northern Territory | 95.5 | ND |
| South Australia | 65.2 | ND |
| Western Australia | 45.5 | ND |
| Victoria | 33.3 | ND |
| New South Wales | 20.0 | ND |
| Chen, 2000 [111] | 15.0 | ND |
| Ngamskulrungroj, 2009 [112] | ND | VGII, VGIII |
| Northern Territory | | |
| Campbell, 2005 [109] | ND | VGI,VGII |
| **New Zealand** | | |
| Chen, 2000 [111] | 4.1 | ND |

ND: No data
